# Diversity and Toxicity of the Genus *Coolia* Meunier in Brazil, and Detection of 44-methyl Gambierone in *Coolia tropicalis*

**DOI:** 10.3390/toxins12050327

**Published:** 2020-05-15

**Authors:** Carlos Eduardo Junqueira de Azevedo Tibiriçá, Manoella Sibat, Luciano Felício Fernandes, Gwenaël Bilien, Nicolas Chomérat, Philipp Hess, Luiz L. Mafra Jr

**Affiliations:** 1 Universidade Federal do Paraná, Centro de Estudos do Mar, Cx, Postal 61, Pontal do Paraná PR 83255-976, Brazil; 2 IFREMER, DYNECO, Phycotoxins Laboratory, 44000 Nantes, France; manoella.sibat@ifremer.fr; 3 Universidade Federal do Paraná, Departamento de Botânica, Cx. Postal 19031, Curitiba PR 81531-990, Brazil; lff@ufpr.br; 4 Ifremer, LITTORAL, Laboratory of Environment and Resources Western Britanny, 29900 Concarneau, France; gwenael.bilien@ifremer.fr (G.B.); nicolas.chomerat@ifremer.fr (N.C.)

**Keywords:** benthic microalgae, toxic dinoflagellates, toxicity assay, cooliatoxin, 44-methyl gambierone

## Abstract

*Coolia* is a genus of marine benthic dinoflagellates which is widely distributed in tropical and temperate zones. Toxicity has been reported in selected *Coolia* species, although the identity of causative compounds is still controversial. In this study, we investigated the taxonomical and toxicological aspects of *Coolia* species from Brazil. Since light- and electron microscopy-based morphology was not enough to distinguish small-celled species, ITS and LSU D1-D3 phylogenetic analyses were used for species definition. Cultures of *Coolia palmyrensis* and *Coolia santacroce* were established from samples collected along the northeastern Brazilian coast, the first record of both species in South Atlantic waters. Cultures of *Coolia malayensis* and *Coolia tropicalis* were also established and exhibited acute *in vivo* toxicity to adults of *Artemia salina*, while *C. palmyrensis* and *C. santacroce* were non-toxic. The presence of 30 yessotoxin analogues, 7 metabolites of *Coolia* and 44 *Gambierdiscus* metabolites was screened in 14 strains of *Coolia*. 44-methyl gambierone (formerly referred to as MTX3) and a new isomer of this compound were detected only in *C. tropicalis*, using both low- and high-resolution LC-MS/MS. To our knowledge, this is the first report of gambierone analogues in dinoflagellates other than *Gambierdiscus*; the role of *C. tropicalis* in ciguatera poisoning thus deserves to be considered in further investigations.

## 1. Introduction

The marine benthic dinoflagellates are recognized for producing a multitude of toxic compounds [1]. Studies concerning these microalgae have increased since the 1970s, after their identification as causative agents of ciguatera poisoning (CP), the most common non-bacterial intoxication affecting human consumers of seafood in the world [2]. Today, ciguatoxins (CTXs) and, to a lesser degree, maitotoxins (MTXs) are regarded as the responsible compounds for CP symptoms [3,4,5]. These toxins, as well as gambieric acids [6], gambierol [7], gambieroxide [8], and gambierones [9], are produced by the dinoflagellate genus *Gambierdiscus*. However, other benthic dinoflagellates may also pose a threat marine organisms and human health, through the production of toxins such as palytoxin and analogues by *Ostreopsis* spp. [2], okadaic acid and dinophysistoxins by *Prorocentrum* spp. [10], and amphidinols by *Amphidinium* spp. [11,12]. Toxicity has also been reported for the broadly distributed genus *Coolia* [13], although the identity of causative compounds is still unresolved in this case.

*Coolia* is present in both tropical and temperate seas [14], and to date, eight species have been described, namely *C. monotis*, *C. tropicalis*, *C. areolata*, *C. canariensis*, *C. malayensis*, *C. palmyrensis*, *C. santacroce* and *C. guanchica* [15]. Before 1995, *Coolia monotis* was the only species of the genus, when *C. tropicalis* was described on a morphological basis [16] and later re-described with molecular support [17]. Molecular techniques are also useful for distinguishing morphologically closely related species such as *C. monotis*, *C. malayensis*, *C. palmyrensis* and *C. santacroce* [18,19], which could be otherwise misidentified [1,20]. In contrast, no molecular data are available for *C. areolata* yet, but this species can be easily identified using morphological features [1]. Therefore, integrative taxonomy should preferably be used for correct identification of *Coolia* spp., and future studies should focus on clarifying morphological differences among closely related species.

The most widely distributed species within the genus is *C. malayensis*, found in every ocean on both tropical and temperate zones [14,21,22]. Other species like *C. tropicalis*, *C. canariensis* and *C. palmyrensis* are present in both Atlantic and Pacific oceans, but they are likely restricted to tropical areas [19,22,23,24]. The reported geographical distribution of the remaining species is much more limited. For instance, *C. monotis* has only been reported in the Atlantic Ocean [14,24], *C. santacroce* in the U.S. Virgin Islands [19] and *C. guanchica* in the Canary Islands [15]. *C. areolata* was cited only from the Indian Ocean [25]. In Brazil, thus far, *C. malayensis* can be found along the entire coast, while *C. tropicalis* and *C. canariensis* are restricted to warmer, northern waters [12,26,27,28].

In an earlier study, *Coolia* sp. (reported as *C. monotis*) exhibited positive effects on hemolytic assays [29] and, since then, this dinoflagellate genu has been considered potentially toxic to marine organisms [1]. Later studies confirmed the toxicity in strains of *C. tropicalis*, *C. malayensis*, *C. palmyrensis* and *C. santacroce* [19,27,30,31,32], although no toxic effects have been reported in *C. monotis*, *C. canariensis*, *C. guanchica*, and in other strains of *C. malayensis* [15,19,33,34]. Furthermore, toxic compounds, named cooliatoxins, have been described from *C. tropicalis* [30] and *C. malayensis* [32], but their chemical identities remain unclear [13]. In view of the marked species-specific differences in toxicity and the inconsistent findings described above, any local assessment of environmental risks associated to the presence of *Coolia* must be preceded by a proper characterization of the genus diversity, as well as a comprehensive screening for individual, toxic compounds and toxic effects among different species.

In the present study, we investigated the phylogeny, morphology, toxin production and toxicity of *Coolia* species from different coastal areas in Brazil, including samples from poorly explored sites. Molecular analysis and electron microscopy were carried out for species identification. As a result, the distribution range of *C. malayensis*, *C. palmyrensis*, *C. santacroce* and *C. tropicalis* was expanded, and toxic and non-toxic strains were characterized and differentiated. We evaluated the toxicity of these four species through acute exposure assays with adults of the microcrustacean *Artemia salina*. Moreover, 14 different monoclonal cultures were screened using either low and high mass spectrometry for the presence of several toxic compounds, including cooliatoxins, yessotoxins, ciguatoxins, maitotoxins, gambieric acids, gambierones, gambierol and gambieroxide.

## 2. Results

### 2.1. Phylogenetics

Species identification was initially evaluated by phylogenetic analyses based on ITS region (ITS 1, 5.8S rDNA and ITS 2) and partial LSU rDNA (D1–D3 domains). The ITS phylogenetic analyses comprised 53 sequences, including 11 sequences from our monoclonal cultures, three outgroup sequences, and sequences retrieved from GenBank. The final ITS alignment was 336-base pairs long. The best-fit model was a TrN (Tamura-Nei model), with base frequencies of A = 0.27370, C = 0.14067, G = 0.22437, T = 0.36125, assuming a gamma distribution shape (G = 0.850). For LSU D1-D3, the final alignment comprised 65 sequences with 745 base pairs. The best-fit model was also a TrN, with base frequencies of A = 0.29774, C = 0.15203, G = 0.23614, T = 0.31410, assuming gamma distribution shape (G = 0.708).

**Figure 1:**
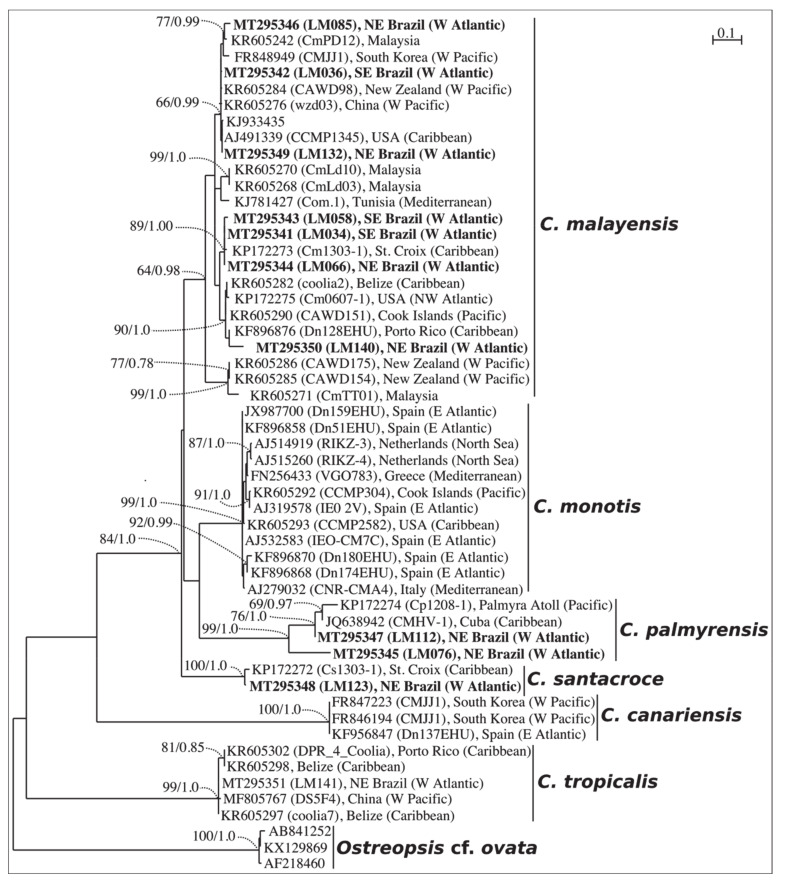
Maximum Likelihood phylogenetic tree inferred from ITS 1, 5.8S rDNA and ITS 2 sequences of various *Coolia* strains (LM034–LM141). *Ostreopsis* cf. *ovata* is used as outgroup. Black vertical bars show distinct *Coolia* clades. Numbers at nodes indicate bootstrap support values from Maximum Likelihood (ML) and posterior probabilities from Bayesian Inference (BI).

**Figure 2:**
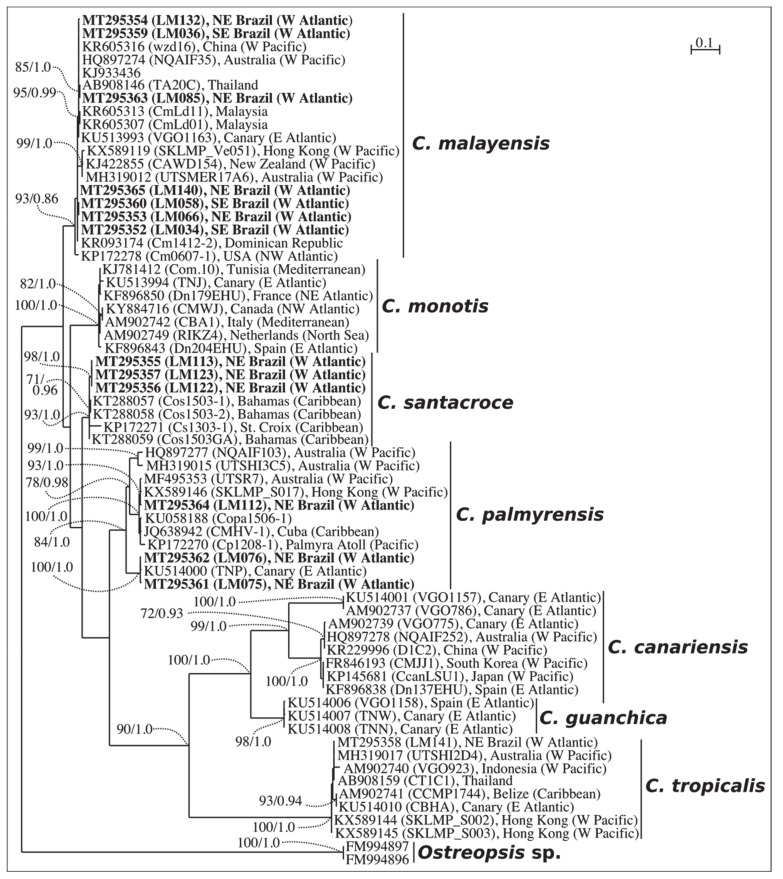
Maximum Likelihood phylogenetic tree inferred from LSU D1–D3 sequences of various *Coolia* strains (LM034–LM141). *Ostreopsis* sp. is used as outgroup. Black vertical bars show distinct *Coolia* clades. Numbers at nodes indicate bootstrap support values from Maximum Likelihood (ML) and posterior probabilities from Bayesian Inference (BI).

Phylogenetic analyses were performed with two reconstruction methods: maximum likelihood (ML) and Bayesian inference (BI). Considering that both ML and BI analyses gave the same tree topology and relationships among clades, only the majority-rule consensus tree of the ML analysis is shown herein. Six distinct clades were found in the phylogeny inferred from ITS sequences (*C. malayensis*, *C. monotis*, *C. santacroce*, *C. palmyrensis*, *C. canariensis* and *C. tropicalis*), and seven clades from LSU D1–D3 (same clades as those inferred from ITS plus *Coolia guanchica*) ( Figure 1 and Figure 2). The sequences from Brazil fit within four (*C. malayensis*, *C. santacroce*, *C. palmyrensis* and *C. tropicalis*) out of the seven described species containing molecular data.

### 2.2. Morphology and Geographical Distribution

Cells of all *Coolia* species evaluated in the present study were nearly spherical, with slightly different degrees of anteroposterior compression among species. Living cells contained many golden-brown chloroplasts. The thecal plate formulae followed a Po, 3’, 7’’, 5’’’, 2’’’’ pattern (according to [1]).

**Figure 3:**
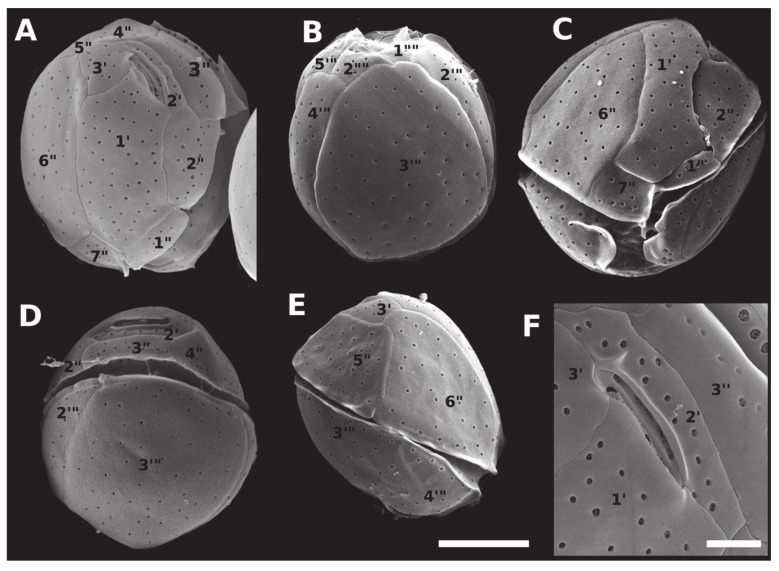
Scanning electron micrographs (SEM) of *Coolia malayensis* (strain LM-036) cells showing: (**A**) apical view; (**B**) antapical view; (**C**) ventral view; (**D**) dorsal view; (**E**) right side view; (**F**) apical pore complex. Scale bar = 10 µm, except in (**F**) (7.5 µm).

**Figure 4:**
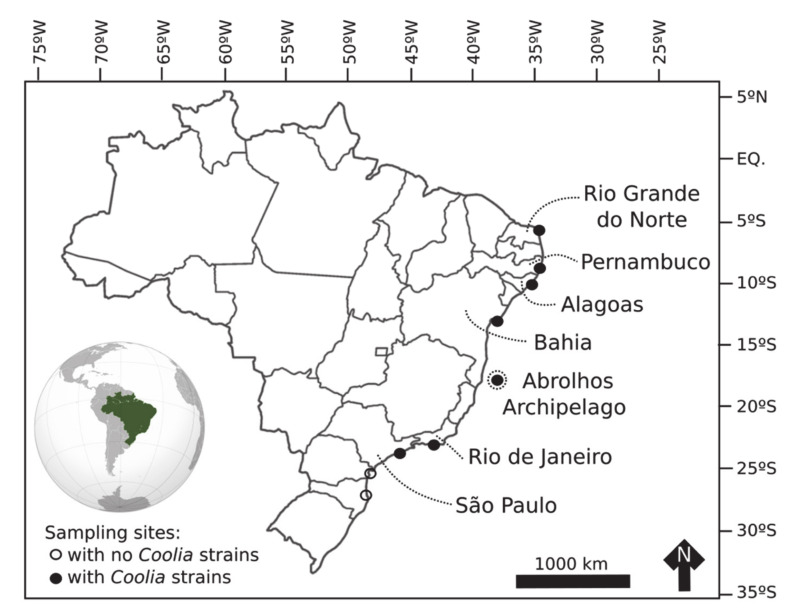
Sampling sites from the present study. Brazilian states and Abrolhos Archipelago (Bahia), where *Coolia* strains were established from, are indicated.

Cells of *C. malayensis* were 23.3 to 29.8-μm deep (dorso-ventral length, DV) (mean ± standard deviation (SD) = 26.7 μm ± 1.9, n = 26), 19.6 to 29.3 μm wide (W) (24.3 ± 3.0, n = 13) and 16.6 to 25.3 μm long (22.0 ± 2.6, n = 15) (antero-posterior length, AP). The DV/W ratio was 0.98–1.37 (1.12 ± 0.15, n = 11). The apical pore plate Po was short, 6.3 ± 0.5 μm, and slightly curved, contiguous to plates 1’, 2’, with plate 3’ dorsally and left displaced, measuring 6.3 ± 0.5 μm (Table 1). The first apical plate (1’) was oblong and hexagonal, with pore density equal to 0.30 pores µm^−1^ (Figure 3; Table 1). The largest plate in apical view was the sixth pre-cingular plate (6’’), touching plates 1’, 3’, 5’’, and 7’’, with pore density of 0.24 pores µm^−1^ (Figure 3; Table 1). The seventh pre-cingular plate (7’’) was small and quadrangular, with 4–9 pores (Table 1). The large third post-cingular plate (3’’’) could be fully viewed in antapical view, exhibiting pore density of 0.18 pores µm^−1^ (Figure 3; Table 1) and occupying most of the hypotheca (Figure 3). The second antapical plate (2’’’’) was small and triangular, containing 3–8 pores (Table 1). The thecal surface was smooth and the mean diameter of thecal pores, 0.33 ± 0.04 μm (n = 36). Strains of *C. malayensis* were obtained from material collected in the following Brazilian States: São Paulo, Rio de Janeiro, Bahia, Alagoas and Rio Grande do Norte (Table 1, Figure 4).

**Figure 5:**
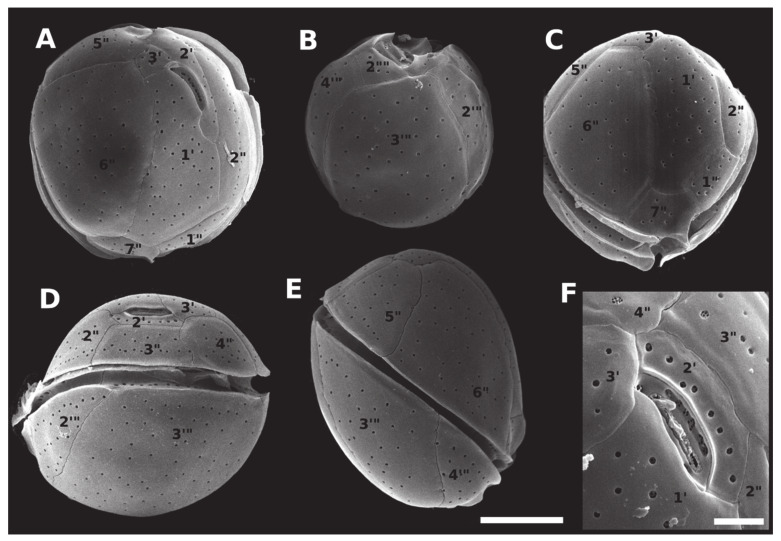
Scanning electron micrographs (SEM) of *Coolia santacroce* (strain LM-113) cells, showing: (**A**) apical view; (**B**) antapical view; (**C**) apical/ventral view; (**D**) dorsal view; (**E**) right side view; (**F**) apical pore complex. Scale bar = 10 µm, except in (**F**) (7.5 µm).

Cells of *C. santacroce* measured 24.0–30.7 μm in DV (27.6 ± 1.7, n = 30), 23.2–29.7 μm in W (26.5 ± 1.8, n = 26) and 18.0–30.0 μm in AP (23.8 ± 3.1, n = 16). The DV/W ratio was 0.98–1.21 (1.05 ± 0.06, n = 20). The mean diameter of thecal pores was 0.32 μm (SD = 0.04, n = 40) and the apical pore plate was 5.9 ± 0.8 μm long (Table 1). The mean pore density was 0.29 pores µm^−1^ for plate 1’, 0.25 pores µm^−1^ for plate 6’’, and 0.22 pores µm^−1^ for plate 3’’’ (Table 1). Cells of *C. santacroce* had 4–10 pores in plate 7’’, and 4–9 pores in plate 2’’’’ (Table 1). Like in *C. malayensis*, size of the plate 3’’’ was impossible to measure in *C. santacroce*, due to the cell curvature (Figure 3 and Figure 5). Specimens originating the *C. santacroce* strains used in the present study were sampled in Abrolhos Archipelago, Bahia (Table 1, Figure 4).

**Figure 6:**
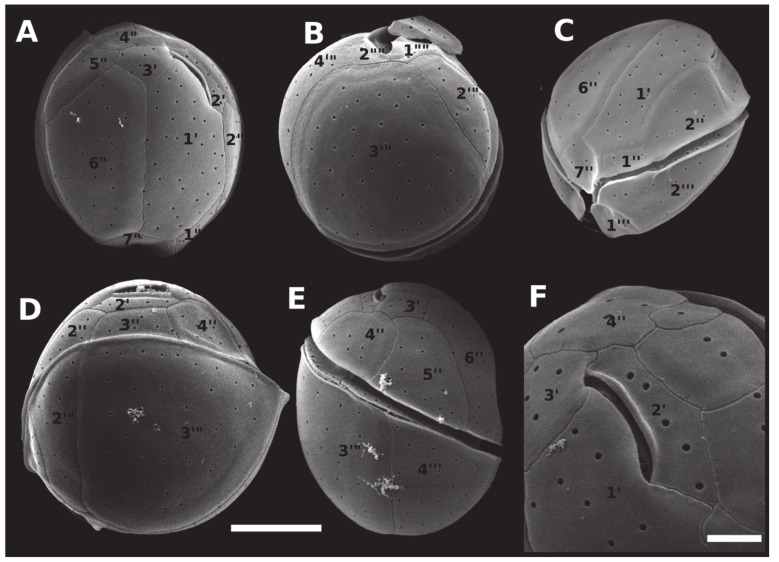
Scanning electron micrographs (SEM) of *Coolia palmyrensis* (strain LM-076) cells showing: (**A**) apical view; (**B**) antapical view; (**C**) apical/ventral view; (**D**) dorsal view; (**E**) right side view; (**F**) apical pore complex. Scale bar = 10 µm, except in (**F**) (7.5 µm).

Mean cell size of *C. palmyrensis* was 19.1–28.4 μm DV (24.1 ± 2.3, n = 27), 17.4–27.3 μm W (22.2 ± 2.4, n = 8) and 16.9–26.1 μm AP (21.0 ± 3.3, n = 8) AP. The DV/W ratio ranged from 0.97 to 1.29 (1.11 ± 0.10, n = 22). The mean diameter of thecal pores was 0.27 ± 0.04 μm (n = 31) and the apical pore plate was 6.3 ± 0.7 μm long (Table 1). Plate 1’ exhibited 0.24 pores µm^−1^, plate 6’’, 0.18 pores µm^−1^, and plate 3’’’, 0.16 pores µm^−1^ (Table 1). Plate 7’’ was ornamented with 3–5 pores, and plate 2’’’’, with 2-7 pores (Table 1). Cells of *C. palmyrensis* exhibited lower mean pore density when compared with *C. malayensis* and *C. santacroce* (Figure 3, Figure 5 and Figure 6), however, some cells were found containing a greater number of pores, so that an overlapping in pore density and pore number was recorded among the three species (Table 1). Strains of *C. palmyrensis* were obtained from samples collected in the coast of Pernambuco State and from Abrolhos Archipelago (Table 1, Figure 4).

**Figure 7:**
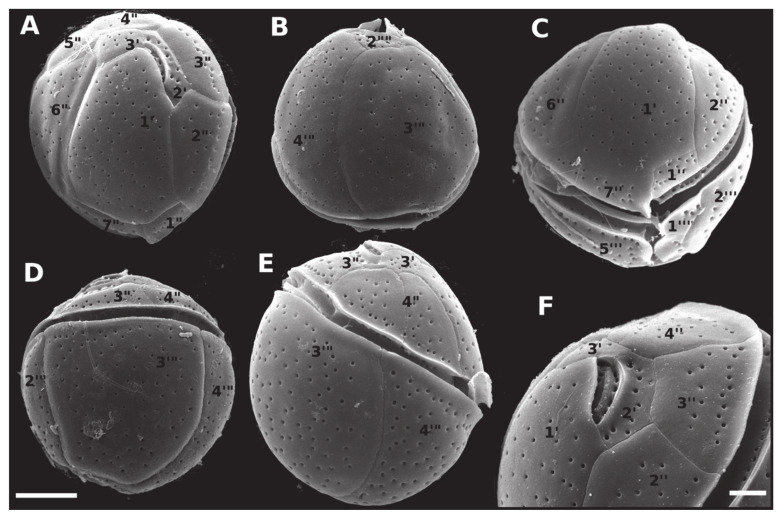
Scanning electron micrographs (SEM) of *Coolia tropicalis* (strain LM-141) cells showing: (**A**) apical view; (**B**) antapical view; (**C**) ventral view; (**D**) dorsal view; (**E**) right side view; (**F**) apical pore complex. Scale bar = 10 µm, except in (**F**) (7.5 µm).

Cells of *C. tropicalis* were 24.3–39.8 μm in DV (34.1 ± 3.3, n = 29), 23.6–39.7 μm in W (32.9 ± 3.2, n = 29) and 25.6–31.3 μm in AP (28.0 ± 2.1, n = 9). The DV/W ratio was 0.97–1.24 (1.08 ± 0.07, n = 20). The mean diameter of thecal pores was 0.35 ± 0.04 μm (n = 31) and the apical pore plate was 7.2 ± 0.8 μm long (Table 1). The mean pore densities were 0.23 pores µm^−1^ for plate 1′, 0.22 pores µm^−1^ for plate 6’’, and 0.22 pores µm^−1^ for plate 3’’’ (Table 1). Cells of *C. tropicalis* had 7–15 pores in the plate 7’’ and 8–12 pores in the plate 2’’’’ (Table 1). This species was easily distinguished from the other three by its elongated, rectangular plate 7’’ (Figure 7). The only successful cultivated strain of *C. tropicalis* was obtained from material collected in Pernambuco (Table 1, Figure 4).

### 2.3. Toxicity

Cells Toxicity was evaluated by feeding adults of *Artemia salina* with increasing biomass of *Coolia* spp. and proportionally decreasing biomass of a nontoxic algal species (*Tetraselmis* sp.) In control treatments, all *A. salina* individuals fed only with *Tetraselmis* sp. survived after 96 h of experiment, with no signs of impaired swimming or any other visual alteration.

**Figure 8:**
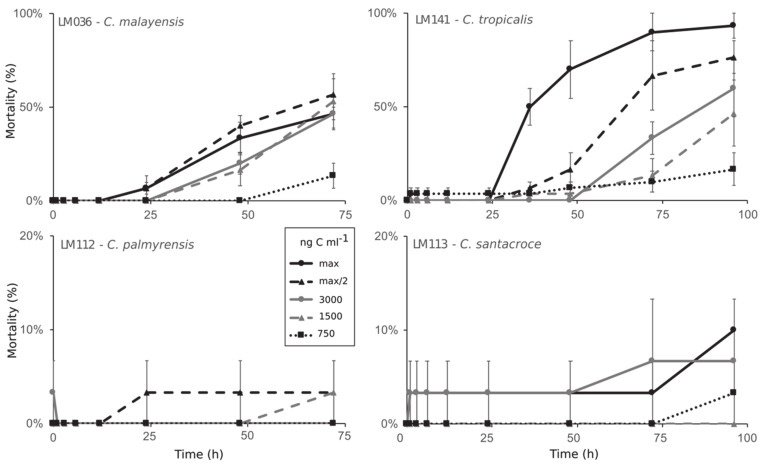
Lethality (%) of *Coolia* spp. to adults of *Artemia salina* over 96 h of exposure. Data series represent different biomass of the toxic algae, expressed as nanograms of carbon per mL. Maximum biomass (max) tested was 19,300 ng C mL^−1^ for *C. malayensis*, 16,000 ng C mL^−1^ for *C. tropicalis*, 15,000 ng C mL^−1^ for *C. santacroce*, and 11,500 ng C mL^−1^ for *C. palmyrensis*. Half of the maximum biomass was also tested (max/2), as well as three fixed biomasses: 3000, 1500 and 750 ng C mL^−1^.

**Figure 9:**
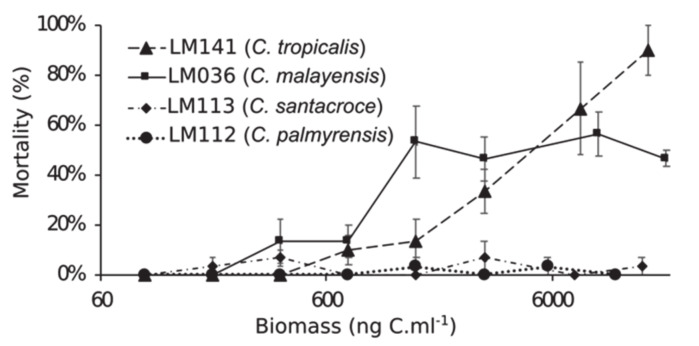
Comparative results of *Coolia* toxicity assays on adults of *Artemia salina*, expressed by the lethal effect (%) after 72 h. Data series represent different species/strains tested.

Among individuals fed with increasing biomass of *C. palmyrensis* (strain LM112) or *C. santacroce* (LM113), no significant toxic effect was recorded (Figure 8 and Figure 9). On the contrary, exposure to *C. malayensis* (LM036) and *C. tropicalis* (LM141) was lethal to *A. salina*, with mortality rates directly related to biomass and exposure time (Figure 8C,D). *C. malayensis* killed up to 57% of *A. salina* individuals after 96 h of experiment, with no differences in mortality rates among those exposed to biomasses of toxic cells equivalent to 1500 ng C mL^−1^ or higher (Figure 8C). *C. tropicalis*, in turn, caused significantly increasing mortality rates with increasing biomass of toxic cells and exposure time, killing > 90% of the individuals after 72–96 h of exposure to the highest biomass tested (16,000 ng C mL^−1^) (Figure 8D). After 24 h, mortality rates caused by *C. tropicalis* were consistently higher than those provoked by equivalent biomass of *C. malayensis* at the same exposure time (Figure 8 and Figure 9).

### 2.4. Toxin Analysis in Coolia spp. Using Low and High Resolution Mass Spectrometry, and Discovery of Gambierone Toxins in C. tropicalis

#### 2.4.1. Screening of Coolia spp. Extracts Using High Resolution Mass Spectrometry (System A)

Fourteen strains of *Coolia* spp. were analyzed in negative (ESI^−^) and positive (ESI^+^) full scan ionization mode on a high-resolution Q-Tof 6550 instrument. Raw data were processed following the Find by Formula (FbF) algorithm in the Agilent MassHunter Qualitative Analysis software, using a Personal Compound Database and Library (PCDL) created by Phycotoxins Laboratory (IFREMER, France). The in-house database used for *Coolia* spp. screening was composed of 81 compounds, including cooliatoxins, yessotoxins, ciguatoxins, maitotoxins, gambieric acids, gambierones, gambierol and gambieroxide (see Supplementary Material, Table S1). Screening with the PCDL allowed identification of compounds based on their formulae and thus detection of the compound itself or isomers.

**Figure 10:**
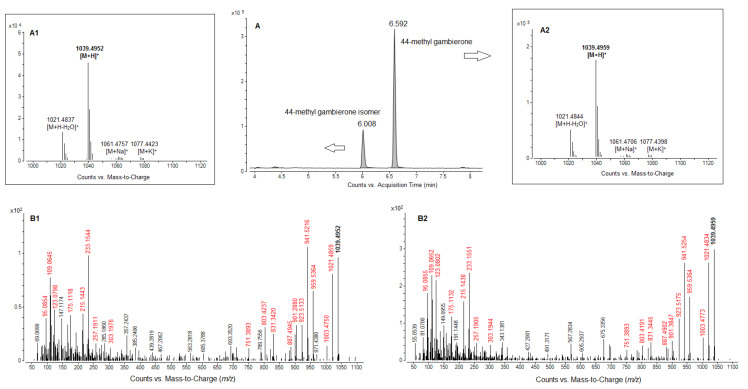
(**A**) LC-HRMS chromatogram (system A) of C. tropicalis extract and high resolution full scan mass spectra acquired in positive mode on the apex of the peak at (**A1**) 6.008 min for 44-methyl gambierone isomer and (**A2**) at 6.592 min for 44-methyl gambierone. HRMS/MS spectra of [M+H]^+^ (*m*/*z* 1039.4931) for (**B1**) 44-methyl gambierone isomer at 6.008 min and for (**B2**) 44-methyl gambierone at 6.592 min, resulting from an average of three collision energies (15, 30 and 45 eV). Fragments common to both compounds were marked in red in the mass spectrum and reported in Table 3.

In ESI^+^ mode, two compounds were tentatively identified in *C. tropicalis* (strain LM141) as 44-methyl gambierone (MTX-3) at 6.0 and 6.6 min retention time (RT), with a score of > 90% and a mass error of < 5 ppm (Figure 10A). The analysis in ESI^−^ mode confirmed the presence of the same two compounds at 6.0 and 6.6 min RT in C. tropicalis, identified as 44-methylgambierone with a score of > 90% and a mass error of < 10 ppm (Supplementary Material, Figure S1). These compounds were additionally investigated in an extract of *Gambierdiscus autrales*, used here as reference, as no analytical standard is available. The peak at RT = 6.6 min assigned to 44-methylgambierone was also present in *G. australes*, but no other peak was detected. The identity of peak at RT = 6.0 min in *C. tropicalis* extract was further determined as a new isomer of 44-methyl gambierone, as detailed below in Section 2.4.2. The assigned positive and negative HRMS ion species for these two compounds are listed in Table 2.

No other known compounds were successfully identified (i.e., with a score > 90% and a mass error < 10 ppm) in any of the other thirteen *Coolia* strains, neither in positive nor in negative mode.

#### 2.4.2. Comparative Fragmentation Between 44-Methyl Gambierone and the New 44-Methyl Gambierone Isomer

As the FbF workflow procedure is not sufficient to unequivocally identify a compound, HRMS/MS spectral acquisition in both ESI^−^ and ESI^+^ mode were necessary to confirm that the new compound was in fact an isomer of 44-methyl gambierone.

In ESI^−^, fragmentation of the molecular anion [M-H]^−^ at *m*/*z* 1037.4785 showed that both molecules shared the same product ions (Table 3, Figure S2). The fragments at *m*/*z* 899.3741 [C43H63O18S]- and 96.9601 [HOSO3], which exhibited very small mass error (Δppm < 3) in our analysis, corresponded to the two fragments also described in previous studies [5,35].

The compound with RT at 6.0 min was further confirmed as an isomer of 44-methyl gambierone, by comparing the positive HRMS/MS with that of 44-methyl gambierone itself (Table 3, Figure 10B). Indeed, the fragmentation pathways showed the formation of protonated fragment ions with a small mass error (Δppm < 5 ppm) at *m*/*z* 959.5330, 941.5216, 923.5133, 905.5012 and 887.4945, corresponding to the sulfite loss followed by successive water losses. Furthermore, similar fragmentation patterns were observed from *m*/*z* 303 to 95 for both compounds, and the gambierone specific fragment ion was detected at *m*/*z* 109.0645, in the isomer as well (Δppm = −2.7).

#### 2.4.3. Quantification of Gambierone Toxins with LC-LRMS/MS (System B) in *C. tropicalis*

**Figure 11:**
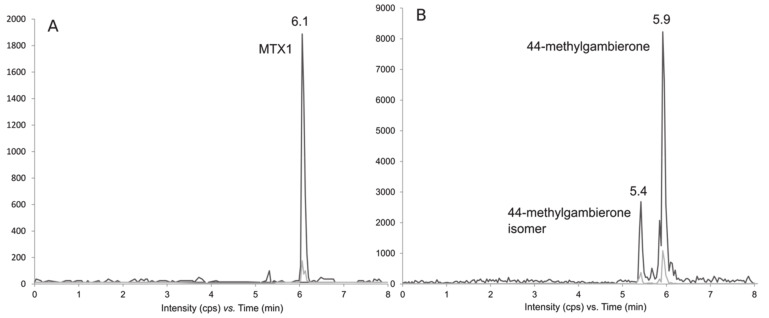
LC-MS/MS Chromatograms acquired in negative MRM mode (system B) of (**A**) MTX1 standard solution at a concentration of 500 ng mL^−1^ (Wako, Japan) and (**B**) 44-methyl gambierone at 5.93 min and the new isomer at 5.42 min in *Coolia tropicalis* extract.

The extract of *Coolia tropicalis* (strain LM141) was further analyzed on a low-resolution API4000 Qtrap instrument, in order to confirm the previous results and to quantify the gambierone toxins (Figure 11). A targeted MRM method was performed in negative ionization mode for the screening of MTXs, gambierones and gambieric acids, as described in Section 5.5.2. Retention times (RTs) were slightly shorter in system B compared to system A, due to differences in dead volumes of the UHPLC systems. The strain of *C. tropicalis* contained 73 and 20 pg MTX-1 eq. cell^−1^ of 44-methylgambierone and 44-methylgambierone isomer, respectively. Therefore, under the culture conditions described in Section 5.1, this *C. tropicalis* strain contained 3.6-fold greater intracellular amounts of 44-methyl gambierone than those of its isomer.

## 3. Discussion

### 3.1. Taxonomy and Phylogeny of Coolia Species

*Coolia* is usually considered a harmful genus of benthic dinoflagellates [1], even though marked species-specific differences in toxicity are observed, as reported herein and discussed later in Section 3.3. Therefore, accurate taxonomy is desirable before any toxicological discussion concerning *Coolia* spp. For instance, prior to the description of *C. malayensis*, and without performing genetic analysis, the occurrence of toxic (CAWD77) and non-toxic (CAWD39 and CCMP304) strains of *C. monotis* was reported in New Zealand [31]. However, by retrieving genetic sequences of the same strains from GenBank, it is possible to find out that two out of three strains belonged, in fact, to *C. malayensis*, and that the actual *C. monotis* strain (CCMP304) was non-toxic. In the present study, before screening our strains for the presence of toxic compounds and toxic effects, we thus assessed *Coolia* spp. taxonomy based on morphology and genetic data, with special focus on the two most toxic species, i.e., *C. malayensis* and *C. tropicalis*.

From all eight species of *Coolia* described so far, only one (*C. areolata*) has no molecular data available to date [15]. Genetic separation among the species is, in general, well resolved. Despite several studies suggesting that *C. malayensis* and *C. monotis* might be the same species [1,37], currently the separation into two different species is more widely accepted [14,19]. In the present study, the phylogenetic analysis on Brazilian strains of *Coolia* spp. revealed similar topology to previous investigations, clearly separating all species based on both ITS (ITS-1, 5.8S rDNA, ITS-2) and LSU (D1-D3) sequences. However, these two DNA fragments resulted in distinct relative distances separating both *C. palmyrensis* and *C. canariensis* from the other species. Thus, we strongly recommend that phylogenetic analyses of *Coolia* include at least two markers in order to confirm species identification.

As previously described, *Coolia* species can be morphologically separated into two major groups based on the shape of the seventh pre-cingular plate (7’’) [14,15,28]. *Coolia monotis*, *C. malayensis* (Figure 3C), *C. palmyrensis* and *C. santacroce* possess a short quadrangular (sometimes triangular) 7’’ plate, and the suture 7’’/1’ is short [14,19]. In contrast, *Coolia tropicalis* (Figure 7C), *C. canariensis*, *C. guanchica* and *C. areolata* exhibit a rectangular 7’’ plate. In this case, the suture between 7’’ and the first apical plate (1’; 7’’/1’) is elongated [15,16,23,25].

The species group encompassing *C. tropicalis* is characterized by a larger cell size compared to the *C. monotis* group (present study, Table 1; [15,16,18,19]). Both species groups are also distinguishable phylogenetically, based on the LSU D1-D3 alignment dataset (see Figure 2). Furthermore, *C. tropicalis* is easily separated from the other species within its own group (i.e., those possessing a rectangular 7’’ plate), based on the smooth cell surface on both epitheca and hypotheca (present study, Figure 7A–B; [15,23,28]).

Conversely, species within the *C. monotis* group are difficult to separate from each other based solely on morphological features. Plate arrangements are quite similar, size ranges overlap, and thecal plates lack any kind of perforation patterns in these smaller-celled species. For *C. malayensis*, a larger third post-cingular plate (3’’’) is the most noticeable feature distinguishing it from other species from *C. monotis* group. In fact, we did observe this feature in most of the cells examined in this study. However, its use as diagnostic of *C. malayensis* may be questionable, as plate 3’’’ can be, in some cases, similar in size to plate 4’’’ (present study, [27,38]). As an additional complicating issue, measuring a large plate like 3’’’ can be inaccurate due to the pronounced cell curvature, making it inappropriate to base cell identification on morphometrics in this case. Alternatively, each of the species from *C. monotis* group could be differentiated from *C. malayensis*, based on a particular feature. *C. palmyrensis*, for instance, may be easily separated from other species based on the density and number of pores on the cell surface [19]. In the present study, we confirmed that *C. palmyrensis* had a lower pore density than its sister species *C. malayensis* and *C. santacroce* (Table 1). However, as previously reported [19], some variability exists, and the ranges of both pore numbers and density can overlap between species. In this sense, *C. malayensis* may sometimes possess low pore density, thus mimicking *C. palmyrensis*, and vice versa (Table 1). Such inconstant thecae features can be attributed to natural variability inside the population, deformation due to culture conditions or to the cell-cycle phase [38]. In conclusion, as already indicated for *C. monotis* and *C. santacroce* [20], species identification within this group of species (*C. monotis* group) should always be supported by genetic data, as otherwise, it can lead to misidentification.

### 3.2. Species Distribution and Diversity in Brazil

Until quite recently, the distribution of benthic dinoflagellates was poorly documented in Brazilian waters, with the genus *Coolia*, represented at that time by *C. malayensis*, found exclusively along the southeastern coast [21]. Recent studies, however, have documented a larger number of species (*C. malayensis*, *C. tropicalis* and *C. canariensis*), occurring over a wider geographical distribution, including the south [12], southeast [26,28] and northeast [27,28] sectors of the Brazilian coast. In the present study, we reported for the first time the species *C. santacroce* and *C. palmyrensis* occurring in South Atlantic waters, and confirmed the high genetic diversity of this genus in Brazil, as suggested by Nascimento et al. [28] Similar species composition has been found in the Canary Islands [15,23], suggesting a high degree of connectivity over the Atlantic Ocean.

The most widely distributed species in Brazil is *C. malayensis*, present along the entire coast (present study, [12,26,27,28]). The other toxic species reported herein, *C. tropicalis*, is more restricted to the northeastern warm waters (present study) and the tropical oceanic island of Trindade [28]. The other three species, *C. canariensis* [28], *C. santacroce* and *C. palmyrensis* (present study), were found exclusively in offshore sites: Trindade Island for *C. canariensis*, Abrolhos Archipelago for *C. santacroce* and *C. palmyrensis*, and an offshore diving site in Pernambuco State for *C. palmyrensis*. Similar patterns of species distribution have been observed in Australia [22] and in the Iberian Peninsula [24], with *C. malayensis* more broadly distributed, *C. tropicalis* restricted to warmer waters, and *C. palmyrensis* present in offshore sites. *Coolia santacroce*, in turn, had only been recorded, so far, in the Caribbean Sea (GenBank sequences). Thus, a general pattern of *Coolia* spp. distribution seems to arise from this and previous studies: *C. malayensis* is globally distributed (see [14]); *C. monotis* is probably broadly distributed in coastal waters of Europe and the East Atlantic [24]; while distributions of *C. tropicalis*, *C. canariensis*, *C. santacroce* and *C. palmyrensis* are more restricted, mainly to warmer waters and/or to offshore sites with lower hydrodynamics (present study, [22]).

### 3.3. Toxicity and Toxin Production

Toxicity within the genus *Coolia* (as *Coolia* sp.) was first reported in the early 1980s, based on hemolytic activity via in vitro assays, although no toxicity to mice and fish was registered for the same methanol extract [29]. Since then, this genus of benthic dinoflagellates has been considered potentially toxic [1]. However, later studies did not detect any toxic activity in several strains/species, using different cell models and organisms. For instance, ethanol extracts of cell pellets of *C. monotis* (strain CCMP304) or *C. malayensis* (CAWD39) were not toxic to mice following intraperitoneal injections in mouse bioassays (MBA) [31]. Additionally, Penna et al. [33] tested methanol extracts of *C. monotis* (strain CM2V) and *C. malayensis* (strain CCMP1345), and did not find any hemolytic activity to human erythrocytes. Similarly, methanol extracts of other *C. monotis* strains (CCMP2582 and CCMP304) exhibited no cytotoxicity to *Rhabdomyosarcoma* cells derived from bone marrow [19]. Moreover, *Artemia franciscana* nauplii were not affected upon exposure to living cells of *C. monotis* (strains Dn23EHU, DN24EHU) or *C. canariensis* (strains Dn28EHU, Dn29EHU) [34], and no toxic effects were observed in *A. salina* nauplii exposed to filtered medium of *C. guanchica* cultures [15]. In the present study, likewise, no toxic effects were observed in adults of *A. salina* exposed to increasing abundances of living *C. santacroce* or *C. palmyrensis* cells. In contrast, cells of *C. malayensis* and *C. tropicalis* were lethal to the micro-crustaceans at equivalent biomass ranges (see Figure 8), confirming the marked species-specific variability in *Coolia* toxicity.

After the early study by Nakajima et al. [29], Holmes et al. [30] evaluated the toxicity of *C. tropicalis* (as *C. monotis*) using MBA. The authors reported mouse mortality caused by the butanol-soluble fraction of the extract, but no toxicity from either hexane- or water-soluble fractions. Later on, acetone and ethanol extracts from two different *C. malayensis* strains (CAWD77 and CAWD151) were also lethal to mice via MBA [31,39]. Moreover, *C. malayensis* (methanol extracts) exhibited the strongest cytotoxic effects to *Rhabdomyosarcoma* cells when compared to *C. santacroce* (intermediate toxicity) and *C. palmyrensis* (low toxicity) [19]. Finally, methanol extracts from Brazilian strains of both *C. malayensis* (UFBA044) and *C. tropicalis* (UFBA055) showed hemolytic activity to sheep erythrocytes [27]. These are the same species here reported as lethal to *A. salina* upon short-term (24–96 h) exposure to living cells. In our experiments, a northeastern Brazilian strain of *C. tropicalis* (LM141) was relatively more toxic than a *C. malayensis* strain isolated from the southeastern coast (LM036).

Taking the results from this and previous toxicity assessments together, we believe that at least *C. tropicalis* and *C. malayensis* should be considered toxic species. Toxicity of other *Coolia* species may vary geographically and should be more carefully evaluated, perhaps using a combination of different assays. For example, *C. santacroce* and *C. palmyrensis* strains from the Caribbean or the Pacific Ocean (Palmyra Atoll) were reported as cytotoxic by Karafas et al. [19], but strains from the present study were not lethal to *A. salina*. Nevertheless, considering only genetically sequenced strains, *C. monotis*, *C. canariensis* and *C. guanchica* have not shown, up to now, any sign of toxic activity (see discussion above), while the toxicity of *C. areolata* has not been examined [25].

The compounds responsible for the toxic activity in *C. malayensis* and *C. tropicalis* are still controversial. In 1995, an analogue of yessotoxin (YTX), then named cooliatoxin, was described in *C. tropicalis* (as *C. monotis*) using low-resolution LC-MS/MS [30]. The exact molecular structure of that compound, however, was not elucidated. Later on, related, yet unique, compounds with fewer oxygen atoms than cooliatoxin or YTX were detected in *C. malayensis* from Okinawa (Japan), and described as disulphated polyether analogues of YTX based on high-resolution LC-MS/MS [32]. However, YTX analogues (including cooliatoxin) have never been detected again in other *Coolia* spp. cultures (present study, [23]). Besides YTX analogues, our strains of *C. malayensis*, *C. palmyrensis* and *C. santacroce* also lacked any other toxic compound produced by another genus of benthic dinoflagellates, *Gambierdiscus*, including maitotoxins, gambierones, gambieroxide, and gambieric acids. A strain of *C. tropicalis*, however, contained relatively high intracellular levels of 44-methyl gambierone (previously referred to as MTX-3 [35]) and a novel isomer of the same compound. Spectral data presented by Holmes et al. [30] suggest that 44-methyl gambierone was not present in that extract (absence of 1037.5 in the negative ionspray mass spectrum), suggesting either misidentification of species, intra-specific variability of toxin production or divergence of *C. tropicalis* between the Pacific and Atlantic Oceans.

According to Boente-Juncal et al. [5], 44-methyl gambierone exhibits similar biological activities to gambierone and CTX3, leading to the decreased viability of undifferentiated neuroblastoma cells and modified expression of excitatory neurotransmitter receptor subunits. This compound can be produced by diverse *Gambierdiscus* species, mainly by *G. australes*, *G. belizeanus* and *G. polynesiensis*, which produce large amounts (reviewed in Longo et al. 2019). Intra-cellular contents of 44-methyl gambierone ranged from 5.8 to 74.1 pg MTX1 eq. cell^−1^ in *G. polynesiensis* (Longo et al. 2019). In the smaller *C. tropicalis* cells (*G. polynsiensis* is twice the size of *C. tropicalis*, see [40] and Table 1 above), we measured 73 and 20 pg MTX1 eq. cell^−1^ of 44-methylgambierone and 44-methylgambierone isomer. Such surprisingly high toxin levels are especially relevant considering that 44-methyl gambierone may be implicated in the neurological manifestations related to ciguatera poisoning (CP) in humans [5]. Thus, the role of *C. tropicalis* as another causative agent of CP deserves to be considered in further investigations.

## 4. Conclusions

*Coolia* is a potentially toxic marine dinoflagellate genus, with many taxonomical and toxicological issues yet to be evaluated and resolved. The smaller-celled *Coolia* species, including *C. malayensis* and similar species, cannot be clearly distinguished from each other based only on morphological features. Thus, in studies of any strain from the *C. monotis* species group (*C. monotis*, *C. malayensis*, *C. santacroce* and *C. palmyrensis*), the use of molecular data is mandatory. *C. malayensis* has proved to be the most broadly distributed species of the genus, found in both temperate and tropical waters of the Atlantic and Pacific Oceans, while other species occur in more restricted areas. This study increased from three to five (out of eight) the number of *Coolia* species reported in Brazilian waters so far, highlighting Brazil as an area of biodiversity for this genus.

Assessment of *Coolia* toxicity can be rather controversial due to the distinct assays/techniques used, but also due to species-specific differences in the capacity of producing toxic compounds. In the present study, *C. malayensis* and *C. tropicalis* cells were toxic to adult Artemia individuals in feeding experiments, while *C. santacroce* and *C. palmyrensis* were not. Using both low- and high-resolution LC-MS/MS, we detected considerable amounts of 44-methyl-gambierone (MTX3)—previously limited to *Gambierdiscus* spp.—and a new 44-methyl gambierone isomer in *C. tropicalis*. According to previous studies, this compound exhibits a powerful cytotoxic effect, which might explain the toxicity in bioassays involving this species.

## 5. Materials and Methods

### 5.1. Sampling and Cultures

Toxicity—Nineteen sampling campaigns were conducted from October 2016 to March 2018, at nine sampling sites along the Brazilian Coast (Figure 4), including coastal rocky shores and islands. Samples were collected and processed following the procedures described in Tester et al. [41] Seaweed samples were vigorously shaken to detach particles, and the seawater containing *Coolia* cells was used for microscopic observation and isolation of living cells. Cells of *Coolia* were isolated using a capillary pipette following successive washing in sterile, local filtered seawater. After initial growth through consecutive cell divisions, the volume of culture was successively doubled by transferring the old aliquot to a larger microplate well, containing an equivalent volume of sterile, 50% diluted f/2 media (f/4), without silica and ~32 salinity. From 10 mL wells, cultures were transferred to 50 and then 250 mL Erlenmeyer flasks, where they were maintained at 26 °C under a 12:12 h light cycle (irradiance of 70 ± 20 μmol m^−2^ s^−1^). Fourteen strains were successfully established and used in the present study (Table 4). For toxin analysis, cultivated cells were harvested at two growth stages (exponential and stationary growth phase). Cells were concentrated by centrifugation (2332 g, 5 min), the supernatant was removed, and samples were stored at −20 °C. Prior to toxin analysis, frozen cell pellets were lyophilized.

### 5.2. DNA Amplification, Sequencing and Molecular Phylogeny

Cultured cells of *Coolia* spp. were harvested by centrifugation (2332 g, 5 min). The supernatant was removed and replaced by ethanol to preserve samples until DNA analysis. Before amplification, single cells from ethanol-preserved samples were isolated with a glass capillary and washed six times with deionized water. Single *Coolia* cells were placed in PCR tubes (at least two tubes for each sample), containing 1–3 µL of deionized water and stored at −20 °C before direct PCR amplifications.

Two consecutive PCR reactions (nested PCR) were performed to amplify the rDNA regions ITS1-5.8S-ITS2 (ITS) and LSU (D1-D3). For the first PCR reaction, 2.5 µL of each primer (ITSfw and D3B, Table 5), 12.5 µL of PCR Master Mix 2X (Promega, Madison^®^, WI, USA) containing the Taq DNA polymerase, dNTPs, MgCl2 and reaction buffers, and 6.5 µL of nuclease-free water were added to each tube. The PCR were performed in a Biometra TOne thermocycler (Analytik Jena), as follows: one initial denaturation step at 95 °C for 2 min, then 35 cycles of 30 s at 95 °C, 1 min at 62 °C (melting temperature, “MT”) and 1 min at 72 °C, and a final elongation step of 5 min 72 °C. For the second PCR reaction, 1 µL of the first product was added to a new tube containing 2.5 µL of each primer (ITSfw and 28S364r for ITS region; D1R and D3B for D1-D3; Table 3), 12.5 µL of GoTaq^®^ G2 Hot Start Green Master Mix (Promega^®^, Madison, WI, USA) and 6.5 µL of nuclease-free water. The second PCR was performed as the first one, changing the MT to 50 °C for ITS, and 56 °C for D1-D3 region. DNA amplifications were controlled by electrophoresis on agarose gel. Positive samples were purified and sequenced, as described in Chomérat et al. [42]

The alignment and phylogenetic analyses were performed as described in Chomérat et al. [42], with modifications as described below. Both ITS and D1–D3 rDNA region datasets were aligned using MAFFT algorithm, with selection of the q-ins-i strategy [43]. Poorly aligned positions were removed using Gblocks algorithm [44], and the most appropriate model of sequence evolution was selected using jModeltest2 v. 2.1.10 [45]. For both rDNA regions, TrN+G were the models used for Maximum Likelihood (ML) and Bayesian Inference (BI) analysis, with 2,000,000 generations performed in BI analysis for both alignments, and sampling every 100 generations. The posterior probabilities of each clade were calculated from the remaining 20,000 trees. For some samples, the primer Coo5.8f (Table 5) was used in the sequencing reaction to obtain clearer sequences from ITS2.

### 5.3. Morphological Observations

Prior to the scanning electron microscopy (SEM) observations, cultured *Coolia* cells were preserved with neutral and acidic lugol (1%). Small sample aliquots (2–5 mL) were placed on a piece of either a 5 µm Millipore filter or a 20 µm plankton net, rinsed with distilled water, and dehydrated in a series of increasing ethanol concentrations (30%, 50%, 70%, 90%, 95% and 100%), followed by critical point drying. Samples were finally mounted on a stub and sputter coated with gold palladium. Cells were observed using a JEOL^®^ JSM 6360-LV (Japan) microscope at 15 Kv. Species identification was based mainly on original and recent *Coolia* spp. descriptions [11,15,16,18].

### 5.4. Toxicity Experiments

The toxicity of selected *Coolia* spp. strains was evaluated through bioassays using adult individuals of the brine shrimp *Artemia salina*. After cyst hatching, *A. salina* larvae were kept in controlled tanks under constant aeration and fed non-toxic *Tetraselmis suecica* cells for 20–30 days. Then, adult specimens of *A. salina* were individually placed in wells of cell culture plates containing 5 mL of autoclaved sea water each. Before each test, plates containing *A. salina* were acclimated for 24–48h under the experimental conditions and fed with *T. suecica* in an amount equivalent to 150 ng C ind^−1^ h^−1^. During the test, they were exposed to increasing cell densities (i.e., treatments) of *Coolia* spp. and a complementary amount of non-toxic *T. suecica* cells, in order to maintain a comparable food supply over all treatments. Cell density of each strain depended on their cell biovolumes, which were calculated from approximate geometrical shapes after measuring 50 cells of each strain [49]. Cell biovolume was then converted into carbon biomass, following conversion factors described in Menden-Deuer et al. [50] The in vivo toxicity assays aimed at providing quantities of *Coolia* spp. equivalent to 4.7, 9.4, 18.8, 37.5, 75 and 150 ng C ind^−1^ h^−1^ for 96 h. The maximum biomass possible (according to each culture cell densities at late exponential growth phase), as well as half of the maximum, were also used as additional treatments. Maximum *Coolia* spp. quantities tested were equivalent to 965, 800, 750, 575 ng C ind^−1^ h^−1^ for *C. malayensis*, *C. tropicalis*, *C. santacroce*, and *C. palmyrensis*, respectively.

Three cell culture plates were used for each experimental treatment, each containing twelve individuals of *A. salina*. From those, ten individuals were exposed to the toxic microalgae and the other two were exposed to the control condition, consisting of non-toxic *T. suecica* cells only. An extra plate containing twelve brine shrimps was used to increase the number of control individuals to 60, while 30 individuals were exposed to each treatment containing *Coolia* cells, adding up to 300 brine shrimps in each experiment. Survival of *A. salina* was evaluated after 1, 3, 12, 24, 48, 72 and 96 h of exposure. Individuals were considered dead if completely motionless at the bottom for 10 consecutive seconds.

### 5.5. Toxin Analysis

Prior to toxin analysis, cell pellets were sonicated in bath ultrasound (Transonic TI-H-15, Elma^®^, Germany) at 45 kHz for 15 min with methanol/water (9:1, v/v). The mixture was centrifuged at 1200 g for 15 min. Supernatant was passed through a centrifuge NanoSep filter (0.2 µm Nylon, PALL^®^, UK) and recovered into plastic vials with conical insert.

Filtered extracts from cell pellets were analyzed using two hybrid systems coupling ultra-high-performance liquid chromatography with tandem mass spectrometry (UHPLC-MS/MS), in either low- (LR) or high-resolution (HR).

#### 5.5.1. System A: HR-MS/MS

System A was composed of a UHPLC system (1290 Infinity II, Agilent Technologies, Santa Clara, CA, USA) coupled to a 6550 ifunnel Q-TOF (Agilent Technologies, CA, USA), equipped with a Dual Jet Stream^®^ ESI source. The high-resolution instrument was operated in both full scan and targeted MS/MS modes. Acquisition was carried out in positive and negative ionization modes, with optimized parameter sources. Temperature was set at 250 °C, drying gas flow at 16 L min^−1^, nebulizer gas at 15 psi and sheath gas at 12 L min^−1^ and 400 °C. Capillary and nozzle voltages were set at 5000 V and 1000 V, respectively. Two reference masses *m*/*z* 121.0509 (purine) and *m*/*z* 922.0099 (hexakis phosphazine) were continuously monitored during the run.

The chromatographic conditions were similar to those described for system B in Section 5.5.2 below, except for the porosity of the Kinetex C18 column used (1.7 µm, instead of 2.6 µm). Mass spectra were acquired from 100 to 1700 *m*/*z*, with an acquisition rate of 2 spectra s^−1^. The targeted MS/MS mode was applied over *m*/*z* 50–1700, with an MS scan rate of 10 spectra s^−1^ and an MS/MS scan rate of 3 spectra s^−1^. Three fixed collision energies were applied by ionization mode (15, 30 and 45 eV in ESI^+^; 30, 45 and 90 eV in ESI^−^), to obtain an overview of the fragmentation pathways. Instrument control, data processing and analysis were conducted using Mass Hunter software v.8.0 (Agilent Technologies, CA, USA).

#### 5.5.2. System B: LR-MS/MS

System B was composed of a UHPLC system (UFLC Nexera, SHIMADZU, Japan), coupled to a hybrid triple quadrupole-linear ion-trap API4000 Qtrap mass spectrometer (ABSciex^®^, Framingham, MA, USA), equipped with a TurboV^®^ electrospray ionization source (ESI). The instrument control, data processing and analysis were conducted using Analyst software 1.6.2 (SCIEX, CA, USA).

A linear gradient using water as eluent A and 95% acetonitrile as eluent B, both eluents containing 2 mM ammonium formate and 50 mM formic acid, was run through a Kinetex C18 column, 50 × 2.1 mm, 2.6 μm, 100 Å (Phenomenex, Torrance, CA, USA). The flow rate was 0.4 mL min^−1^, the injection volume was 5 μL and the column temperature was 40 °C. The elution gradient was set as follows: 10% B to 95% B from 0 to 10 min, hold at 95% B for 2 min, decrease from 95% to 10% in 1 min and hold during 3 min to equilibrate. Mass spectrometric detection was performed in negative ionization mode using MRM scanning. The *m*/*z* transition used is listed in Table 6 for *Gambierdiscus* metabolites and in Table S2 for YTXs toxins, for which certified standards were available. The optimized ESI^-^ parameters were set as follows: curtain gas at 25 psi, ion spray at −4500 V, turbo gas temperature at 500 °C, gas 1 and 2 at 50 psi, declustering potential at −210 V for *Gambierdiscus* metabolites and −120 V for YTXs toxins and an entrance potential at −10 V.

#### 5.5.3. Quantification of Gambierone Toxins in *C. tropicalis*

In order to quantify the MTX and gambierone toxins, a calibration curve of MTX1 was prepared from successive dilutions of a standard solution (Wako, Japan) in 50% MeOH, with concentrations ranging from 0.2 to 5.0 µg mL^−1^. Due to the lack of analytical standards, each targeted compound was quantified from the MTX1 calibration curve prepared, assuming equivalent molar response. The amounts of gambierone toxins present in the *C. tropicalis* extract were estimated using the MRM transition [M−H]^−^/[M−H]^−^, whereas the bi-charged molecular anion [M−2H]^2−^/[M−2H]^2−^ was used for MTX1. This was necessary, since the singly charged ion of MTX-1 is out of the mass range effectively detected by the instrument (50–2800 Da).

## Figures and Tables

**Table 1:** Cell measurements of *Coolia* spp., in µm, as obtained from scanning electron micrographs (SEM). Minimum and maximum values, as well as the number of cells measured (in italics), are provided in parentheses following each average value. DV = dorso-ventral length (depth); W = width; AP = antero-posterior length (height); APC = apical pore complex. Pore density (pores µm^−1^) was measured using 5 × 5 µm square, placed in the center of the thecal plate.

Measurement		*C. malayensis*	*C. santacroce*	*C. palmyrensis*	*C. tropicalis*
**Cell size**	DV	26.7 (23.3–29.8, *26*)	27.6 (24.0–30.7, *30*)	24.1 (19.1–28.4, *27*)	34.1 (24.3–39.8, *29*)
	W	24.3 (19.6–29.3, *13*)	26.5 (23.2–29.7, *26*)	22.2 (17.4–27.3, *29*)	32.9 (23.6–39.7, *29*)
	AP	22.0 (16.6–25.3, *15*)	23.8 (18.0–30.0, *16*)	21.0 (16.9–26.1, *8*)	28.0 (25.6–31.3, *9*)
DV/W		1.12 (0.98–1.37, *11*)	1.05 (0.98–1.21, *20*)	1.11 (0.97–1.29, *22*)	1.08 (0.97–1.24, *20*)
APC length		6.3 (5.3–7.4, *13*)	5.9 (4.9–7.4, *8*)	6.3 (5.5–7.5, *8*)	7.2 (6.3–8.6, *9*)
Pore size		0.33 (0.26–0.42, *36*)	0.31 (0.25–0.44, *40*)	0.27 (0.23–0.42, *31*)	0.35 (0.27–0.42, *31*)
Pore density	Plate 1’	0.30 (0.20–0.44, *16*)	0.29 (0.20–0.36, *12*)	0.24 (0.20–0.28, *8*)	0.23 (0.16–0.28, *11*)
	Plate 6’’	0.24 (0.16–0.32, *14*)	0.25 (0.20–0.32, *10*)	0.18 (0.16–0.20, *7*)	0.22 (0.16–0.28, *8*)
	Plate 3’’’	0.18 (0.12–0.24, *14*)	0.22 (0.16–0.28, *8*)	0.16 (0.11–0.20, *11*)	0.22 (0.16–0.28, *5*)
Pore number	Plate 7’’	6.8 (4–9, *19*)	6.9 (4–10, *13*)	4.0 (3–5, *6*)	13.3 (7–15, *10*)
	Plate 2’’’’	5.1 (3–8, *13*)	6.1 (4–9, *7*)	3.5 (2–7, *8*)	9.7 (8–12, *7*)
Origin of strains (Brazilian states)		São Paulo, Rio de Janeiro, Bahia, Alagoas and Rio Grande do Norte	Abrolhos Archipelago (Bahia)	Abrolhos Archipelago (Bahia) and Pernambuco	Rio Grande do Norte

**Table 2:** HRMS ion species corresponding to the accurate mono-isotopic *m*/*z* for 44-methyl gambierone and its isomer. Mass differences (Δppm) were compared between measured and exact theoretical mass.

Ion Species (Mono-Isotopic *m*/*z*)	44-Methyl Gambierone	44-Methyl Gambierone Isomer
	RT = 6.6 min	RT = 6.0 min
[M+H]^+^	1039.4959 (−2.1)	1039.4952 (+2.0)
[M+H-H_2_O]^+^	1021.4844 (+0.9)	1021.4837 (+3.3)
[M+Na]^+^	1061.4706 (−2.1)	1061.4757 (+0.4)
[M+K]^+^	1077.4398 (−8.5)	1077.4423 (−6.2)
[M-H]^−^	1037.4797 (+1.1)	1037.4766 (−1.9)
[M-H-H_2_O]^−^	1019.4651 (−2.8)	1019.4587 (−9.1)

**Table 3:** List of assigned HRMS/MS fragment ions for 44-methyl gambierone and its isomer obtained from MS^2^ spectra of [M+H]^+^ at *m*/*z* 1039.4931 for ESI^+^, and of [M-H]^−^ at *m*/*z* 1037.4785 for ESI^−^. The *m*/*z* values correspond to the accurate mono-isotopic *m*/*z*.

Parent or Fragment Ion	Formula	44-methyl Gambierone		44-methyl Gambierone Isomer		Ref.
		(*m*/*z*)	Δppm	(*m*/*z*)	Δppm	
**Parent ion [M+H]** ^+^	**C_52_H_79_O_19_S^+^**	1039.4909	−2.1	1039.4952	+2.0	
**ESI** ^+^ **Fragment ions**	C_52_H_77_O_18_S^+^	1021.4834	+0.9	1021.4859	+3.3	[5]
	C_52_H_75_O_17_S^+^	1003.4773	+5.3	1003.4750	+3.0	
	C_52_H_79_O_16_^+^	959.5364	+0.1	959.5330	−3.4	
	C_52_H_77_O_15_^+^	941.5254	−0.3	941.5216	−4.4	
	C_52_H_75_O_14_^+^	923.5175	+2.5	923.5133	−2.0	
	C_52_H_73_O_13_^+^	905.5004	−4.6	905.5012	−3.7	
	C_52_H_71_O_12_^+^	887.4902	−4.3	887.4945	+0.6	
	C_43_H_65_O_18_S^+^	901.3847	−4.3	901.3880	−0.7	[36]
	C_43_H_63_O_14_^+^	803.4191	−2.7	803.4237	+3.1	
	C_15_H_21_O_2_^+^	233.1551	+6.4	233.1544	+3.4	
	C_15_H_19_O^+^	215.1438	+3.5	215.1443	+5.9	
	C_8_H_11_O^+^	123.0802	−2.0	123.0810	−5.2	
	C_7_H_9_O^+^	109.0652	+3.8	109.0645	−2.7	
	C_7_H_11_^+^	95.0855	−0.3	95.0854	−1.3	
**Parent ion [M-H]** **^−^**	C_52_H_77_O_19_S^−^	1037.4797	+1.1	1037.4766	−1.9	
**ESI^−^** **Fragment ions**	C_43_H_63_O_18_S^−^	899.3743	+0.3	899.3732	−1.0	[35]
	[HOSO_3_]^−^	96.9601	0	96.9599	−2.1	

**Table 4:** *Coolia* spp. strains used in the present study.

Specie	Strain	Brazilian state	Latitude (S)	Longitude (W)	Date
*Coolia malayensis*	LM-034	São Paulo	23°50′36.90″	45°24′15.66″	12/11/2016
*C. malayensis*	LM-036	Rio de Janeiro	23°03′19.20″	44°19′45.42″	10/11/2016
*C. malayensis*	LM-058	Rio de Janeiro	23°01′16.20″	44°19′47.52″	23/01/2017
*C. malayensis*	LM-066	Bahia	12°34′54.30″	38°00′03.90″	11/03/2017
*C. malayensis*	LM-085	Bahia	12°57′20.46″	38°21′36.06″	10/03/2017
*C. malayensis*	LM-132	Alagoas	09°40′07.52″	35°42′45.37″	22/02/2018
*C. malayensis*	LM-140	Rio Grande do Norte	05°33′53.30″	35°04′20.90″	10/03/2018
*Coolia palmyrensis*	LM-075	Pernambuco	08°35′31.02″	34°54′43.02″	28/03/2017
*C. palmyrensis*	LM-076	Pernambuco	08°35′31.02″	34°54′43.02″	28/03/2017
*C. palmyrensis*	LM-112	Bahia (Abrolhos)	18°02′00.00″	38°41′53.88″	15/10/2017
*Coolia santacroce*	LM-113	Bahia (Abrolhos)	18°02′00.00″	38°41′53.88″	15/10/2017
*C. santacroce*	LM-122	Bahia (Abrolhos)	18°02′00.00″	38°41′53.88″	15/10/2017
*C. santacroce*	LM-123	Bahia (Abrolhos)	18°02′53.88″	38°41′53.88″	15/10/2017
*Coolia tropicalis*	LM-141	Rio Grande do Norte	05°33′53.30″	35°04′20.90″	11/03/2018

**Table 5:** Oligonucleotide primers used in the present study.

Primer	Sequence	Reference
ITSfw	5′-GTAGGTGAACCTGCGGAAGG-3ʹ	[46]
Coo5.8f	5′-ATGCAGAATCCCGTGAATCA-3ʹ	Present study
D1R	5′-ACCCGCTGAATTTAAGCATA-3ʹ	[47]
364R	5’-CTCTCTTTTCAAAGTCCTTTTC-3’	Present study
D3B	5′-TCGGAGGGAACCAGCTACTA-3ʹ	[48]

**Table 6:** List of MRM transitions (*m*/*z*) used in ESI- to detect MTXs, gambierone toxins and gambieric acids on system B (API 4000QTrap).

Compound	MRM transitions (*m*/*z*)		CE (eV)	CXP (eV)
MTX1	1689.8 >1689.6	[M-2H]^2−^/[M-2H]^2−^	−40	−15
	1689.8 > 96.9	[M-2H]^2−^/[HOSO_3_]^2−^	−125	−21
	1126.2 > 1126.2	[M-3H]^3−^/[M-3H]^3−^	−40	−15
	1126.2 > 96.9	[M-3H]^3−^/[HOSO_3_]^3−^	−125	−21
MTX2	1637.5 > 1637.5	[M-2H]^2−^/[M-2H]^2−^	−40	−15
	1637.5 > 96.9	[M-2H]^2−^/[HOSO_3_]^2−^	−125	−21
	1091.5 > 1091.5	[M-3H]^3−^/[M-3H]^3−^	−40	−15
	1091.5 > 96.9	[M-3H]^3−^/[HOSO_3_]^3−^	−125	−21
MTX4	1646.2 > 1646.2	[M-2H]^2−^/[M-2H]^2−^	−40	−15
	1646.2 > 96.9	[M-2H]^2−^/[HOSO_3_]^2−^	−125	−21
desulfo-MTX1	1649.8 > 1649.8	[M-2H]^2−^/[M-2H]^2−^	−40	−15
	1649.8 > 96.9	[M-2H]^2−^/[HOSO_3_]^2−^	−125	−21
didehydro-demethyl- desulfo-MTX1	1641.8 > 1641.8	[M-2H]^2−^/[M-2H]^2−^	−40	−15
	1641.8 > 96.9	[M-2H]^2−^/[HOSO_3_]^2−^	−125	−21
Gambierone	1023.5 > 1023.5	[M-H]^−^/[M-H]^−^	−40	−15
	1023.5 > 96.9	[M-H]^−^/[HOSO_3_]^−^	−125	−21
44-methylgambierone	1037.6 > 1037.6	[M-H]^−^/[M-H]^−^	−40	−15
	1037.6 > 96.9	[M-H]^−^/[HOSO_3_]^−^	−125	−21
Gambieroxide	1193.6 > 1193.6	[M-H]^−^/[M-H]^−^	−20	−15
	1193.6 > 96.9	[M-H]^−^/[HOSO_3_] ^−^	−125	−21
Gambieric acid A	1055.1 > 1055.1	[M-H]^−^/[M-H]^−^	−20	−15
	1055.1 > 1037.1	[M-H]^−^/[M-H-H_2_O] ^−^	−40	−15
Gambieric acid B	1069.1 > 1069.1	[M-H]^−^/[M-H]^−^	−20	−15
	1069.1 > 1051.1	[M-H]^−^/[M-H-H_2_O] ^−^	−40	−15
Gambieric acid C	1183.7 > 1183.7	[M-H]^−^/[M-H]^−^	−20	−15
	1183.7 > 1165.7	[M-H]^−^/[M-H-H_2_O] ^−^	−40	−15
Gambieric acid D	1197.7 > 1197.7	[M-H]^−^/[M-H]^−^	−20	−15
	1197.7 > 1179.7	[M-H]^−^/[M-H-H_2_O] ^−^	−40	−15

## Supplementary Materials

The following are available online at https://www.mdpi.com/2072-6651/12/5/327/s1, Table S1. List of compounds extracted from the Personal Compound Database and Library (PCDL, Agilent Mass Hunter software), created by Phycotoxins Laboratory (IFREMER, France) and used for Coolia spp. Screening, Table S2: List of MRM transitions (*m*/*z*) used in ESI- to detect YTXs on system B (API 4000QTrap), Figure S1. (A) LC-HRMS chromatogram of C. tropicalis extract and high resolution full scan mass spectra acquired in negative mode on the apex of peaks at (A1) 6.0 min for 44-methyl gambierone isomer and (A2) at 6.6 min for 44-methyl gambierone, Figure S2. HRMS/MS spectra of [M-H]- (*m*/*z* 1037.4785) for (A) 44-methyl gambierone isomer at 6.0 min and for (B) 44-methyl gambierone at 6.6 min, resulting from an average of three collision energies (40, 65 and 90 eV).
