# Supplementary material for: Diversity and Toxicity of the Genus Coolia Meunier in Brazil, and Detection of 44-methyl Gambierone in Coolia tropicalis
Source: Toxins (Basel). 2020 May 15;12(5):327. doi: 10.3390/toxins12050327 (PMC7290302; doi:10.3390/toxins12050327)
Supplement: Supplementary file 1 [file toxins-12-00327-s001.pdf]

Supplementary Material for: Diversity and Toxicity of the Genus *Coolia* Meunier in Brazil, and Detection of 44-methyl Gambierone in *Coolia tropicalis*

Carlos Eduardo Junqueira de Azevedo Tibiriçá, Manoella Sibat, Luciano Felício Fernandes, Gwenaël Bilien, Nicolas Chomérat, Philipp Hess and Luiz L. Mafra, Jr

Table S1. List of compounds extracted from the Personal Compound Database and Library (PCDL, Agilent Mass Hunter software), created by Phycotoxins Laboratory (IFREMER, France) and used for *Coolia* spp. screening.

Name	Molecular Formula	Mass (Da)	CAS
Cooliatoxin metabolite 1	$C_{56}H_{78}O_{18}S_2$	1102.46296	
Cooliatoxin metabolite 2	$C_{57}H_{80}O_{18}S_2$	1116.47861	
Cooliatoxin metabolite 3	$C_{57}H_{78}O_{19}S_2$	1130.45787	
Cooliatoxin metabolite 4	$C_{57}H_{84}O_{18}S_2$	1120.50991	
Cooliatoxin metabolite 5	$C_{58}H_{86}O_{18}S_2$	1134.52556	
Cooliatin	$C_{15}H_{22}NaO_4$	289.14158	
desulfo-YTX (cooliatoxin)	$C_{55}H_{82}O_{18}S$	1062.52219	255041-59-9
YTX	$C_{55}H_{82}O_{21}S_2$	1142.479	112514-54-2
Homo-YTX	$C_{56}H_{84}O_{21}S_2$	1156.49465	196309-94-1
45-OH-YTX	$C_{55}H_{82}O_{22}S_2$	1158.47392	124863-39-4
45-OH-Homo-YTX	$C_{56}H_{84}O_{22}S_2$	1172.48957	196309-97-4
COOH-YTX	$C_{55}H_{82}O_{23}S_2$	1174.46883	262842-91-1
COOH Homo YTX	$C_{56}H_{84}O_{23}S_2$	1188.48448	292850-13-6
Heptanor-41-oxo-Homo-YTX	$C_{49}H_{74}O_{21}S_2$	1062.4164	346631-41-2
40-epi-Heptanor-41-oxo-YTX	$C_{48}H_{72}O_{21}S_2$	1048.40075	803745-66-6
YTX enone	$C_{48}H_{72}O_{21}S_2$	1048.40075	803745-67-7
Heptanor-41-oxo-YTX	$C_{48}H_{72}O_{21}S_2$	1048.40075	448238-76-4
40-epi-Heptanor-nor-YTX	$C_{45}H_{67}O_{20}S_2$	991.366711	
Heptanor-nor-YTX enone	$C_{45}H_{67}O_{20}S_2$	991.366711	
Heptanor-41-nor-oxo-YTX	$C_{45}H_{67}O_{20}S_2$	991.366711	
32-O-triarabinosyl-HomoYTX	$C_{71}H_{107}O_{33}S_2$	1551.613602	
32-O-diarabinosyl-HomoYTX	$C_{66}H_{99}O_{29}S_2$	1419.571344	
32-O-triarabinosyl-YTX	$C_{70}H_{105}O_{33}S_2$	1537.597952	
9-Me-41-Homo-YTX amide	$C_{61}H_{94}NO_{25}S_2$	1304.555634	
32-O-arabinosyl-YTX	$C_{60}H_{89}O_{25}S_2$	1273.513435	
44,45-diOH-41-Homo-YTX	$C_{56}H_{85}O_{23}S_2$	1189.492305	
44,45-diOH-Homo-YTX	$C_{56}H_{85}O_{23}S_2$	1189.492305	
44,55-diOH-YTX	$C_{55}H_{84}O_{23}S_2$	1176.48448	862783-86-6
9-Me-41-Homo-YTX	$C_{57}H_{85}O_{21}S_2$	1169.502476	
41-Homo-YTX	$C_{56}H_{84}O_{21}S_2$	1156.49465	
Trinor-YTX	$C_{52}H_{78}O_{21}S_2$	1102.4477	181365-95-7
nor-YTX	$C_{52}H_{77}O_{20}S_2$	1085.444961	
32-O-diarabinosyl-YTX	$C_{65}H_{97}O_{29}S_2$	1405.555694	
41-Homo-YTX amide	$C_{60}H_{92}NO_{25}S_2$	1290.539984	
32-O-arabinosyl-HomoYTX	$C_{61}H_{91}O_{25}S_2$	1287.529085	
9-Me-44,45-diOH-41-Homo-YTX	$C_{57}H_{87}O_{23}S_2$	1203.507956	
MTX	$C_{164}H_{258}O_{68}S_2$	3379.61719	59392-53-9
Maitotoxin sodium	$C_{164}H_{256}Na_2O_{68}S_2$	3423.58108	59392-53-9
MTX4	$C_{157}H_{241}NO_{68}S_2$	3292.48724	
Gambierone	$C_{51}H_{76}O_{19}S$	1024.47015	
44-methylgambierone (MTX3)	$C_{52}H_{78}O_{19}S$	1038.4858	
Gambieroxide	$C_{60}H_{90}O_{22}S$	1194.56445	
Gambieric acid A	$C_{59}H_{92}O_{16}$	1056.63854	138434-64-7

Gambieric acid B	$C_{60}H_{94}O_{16}$	1070.65419	141363-65-7
Gambieric acid C	$C_{65}H_{100}O_{19}$	1184.68588	138458-89-6
Gambieric acid D	$C_{66}H_{102}O_{19}$	1198.70153	141363-66-8
Gambierol	$C_{43}H_{64}O_{11}$	756.44486	
P-CTX 1	$C_{60}H_{86}O_{19}$	1110.57634	11050-21-8
P-CTX 2 (52-epi-54-deoxy-P-CTX1)	$C_{60}H_{86}O_{18}$	1094.58142	142185-85-1
P-CTX 3 (54-deoxy P-CTX1)	$C_{60}H_{86}O_{18}$	1094.58142	139641-09-6
P-CTX 3C	$C_{57}H_{82}O_{16}$	1022.56029	148471-85-6
P-CTX 3B	$C_{57}H_{82}O_{16}$	1022.56029	263336-58-9
P-CTX 4B	$C_{60}H_{84}O_{16}$	1060.57594	123676-76-6
P-CTX 4A	$C_{60}H_{84}O_{16}$	1060.57594	66231-73-0
50-OH P-CTX 3	$C_{60}H_{86}O_{19}$	1110.57634	263336-54-5
52-epi P-CTX 1	$C_{60}H_{86}O_{19}$	1110.57634	189013-49-8
54-Epi P-CTX 1	$C_{60}H_{86}O_{19}$	1110.57634	287732-40-5
52,54- Diepi P-CTX 1	$C_{60}H_{86}O_{19}$	1110.57634	287732-42-7
M-seco P-CTX 4A	$C_{60}H_{86}O_{17}$	1078.5865	287412-00-4
M-seco 2,3dihydro, 2-OH-49-O-Me-P-CTX 3C	$C_{58}H_{88}O_{18}$	1072.59707	374624-43-8
2,3-dihydro, 2,3,51-triOH P-CTX 3C	$C_{57}H_{84}O_{19}$	1072.56069	263336-63-6
A-seco-2,3 dihydro,51-OH P-CTX 3C	$C_{57}H_{86}O_{18}$	1058.58142	263336-64-7
2,3 diOH P-CTX 3C	$C_{57}H_{84}O_{18}$	1056.56577	263336-62-5
M-seco 49-O-Me-P-CTX 3C	$C_{58}H_{86}O_{17}$	1054.58651	287411-99-8
2,3- dihydro, 2-oxo,51-OH P-CTX 3C	$C_{57}H_{82}O_{18}$	1054.55012	287732-77-8
2,3- dihydro, 3-oxo, 51-OH P-CTX 3C	$C_{57}H_{82}O_{18}$	1054.55012	263336-61-4
2,3- dihydro-2-OH P-CTX 3C	$C_{57}H_{84}O_{17}$	1040.57086	287732-78-9
M-seco P-CTX 3C	$C_{57}H_{84}O_{17}$	1040.57086	287411-98-7
2,3- dihydro-3-OH P-CTX-3C	$C_{57}H_{84}O_{17}$	1040.57085	263336-60-3
51-OH P-CTX 3C	$C_{57}H_{82}O_{17}$	1038.55521	263336-59-0
3-OH,7-oxo P-CTX	$C_{60}H_{88}O_{21}$	1144.58181	263336-57-8
4- OH, 7-oxo P-CTX	$C_{60}H_{88}O_{21}$	1144.58181	287732-85-8
7-Oxo P-CTX	$C_{60}H_{86}O_{20}$	1126.57125	263336-55-6
C-CTX 1	$C_{62}H_{92}O_{19}$	1140.62329	193363-37-0
C-CTX 2	$C_{62}H_{92}O_{19}$	1140.62329	193363-38-1
I-CTX 1	$C_{62}H_{92}O_{19}$	1140.62329	
I-CTX 2	$C_{62}H_{92}O_{19}$	1140.62329	
I-CTX 3	$C_{62}H_{92}O_{20}$	1156.6182	
I-CTX 4	$C_{62}H_{92}O_{20}$	1156.6182	
I-CTX 5	$C_{62}H_{90}O_{19}$	1138.60763	
I-CTX 6	$C_{62}H_{90}O_{20}$	1154.60255	

Table S2. List of MRM transitions (*m*/*z*) used in ESI$^-$ to detect YTXs on system B (API 4000QTrap).

Compound	MRM transitions (*m*/*z*)	CE (eV)	CXP (eV)
YTX	1141.4 > 1061.6	−48	−17
	1141.4 > 855.6	−98	−19
Homo YTX	1155.6 > 1075.6	−48	−17
	1155.6 > 869.4	−98	−19
45-OH YTX	1157.5 > 1077.5	−48	−17
	1157.5 > 855.5	−98	−19
45-OH homo YTX	1171.5 > 1091.5	−48	−17
	1171.5 > 869.4	−98	−19
COOH YTX	1173.5 > 1093.5	−48	−17
	1173.5 > 855.5	−98	−19
Homo COOH YTX	1187.5 > 1107.5	−48	−17
	1187.5 > 869.4	−98	−19

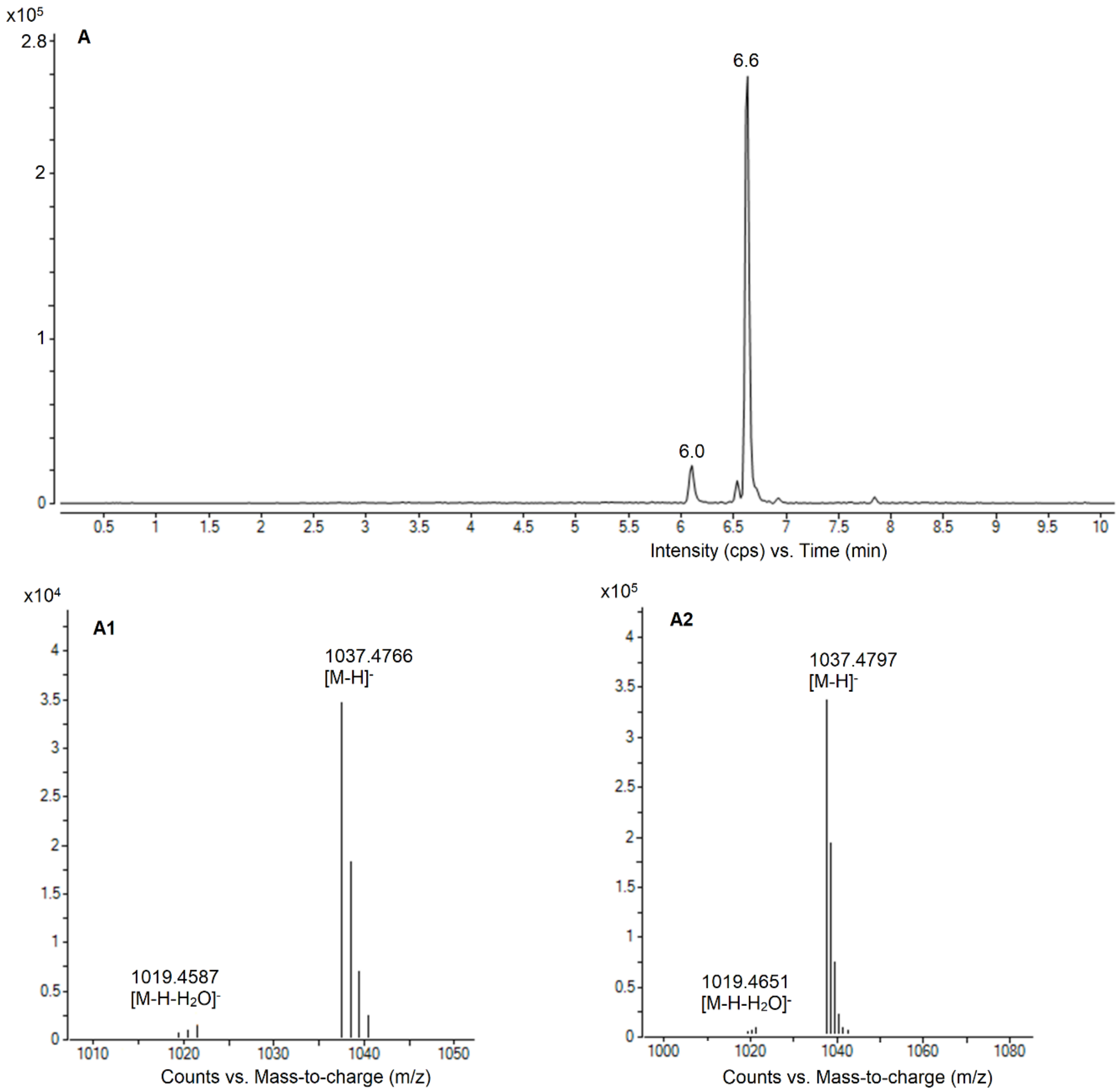

Figure S1. (**A**) LC-HRMS chromatogram of *C. tropicalis* extract and high resolution full scan mass spectra acquired in negative mode on the apex of peaks at (**A1**) 6.0 min for 44-methyl gambierone isomer and (**A2**) at 6.6 min for 44-methyl gambierone.

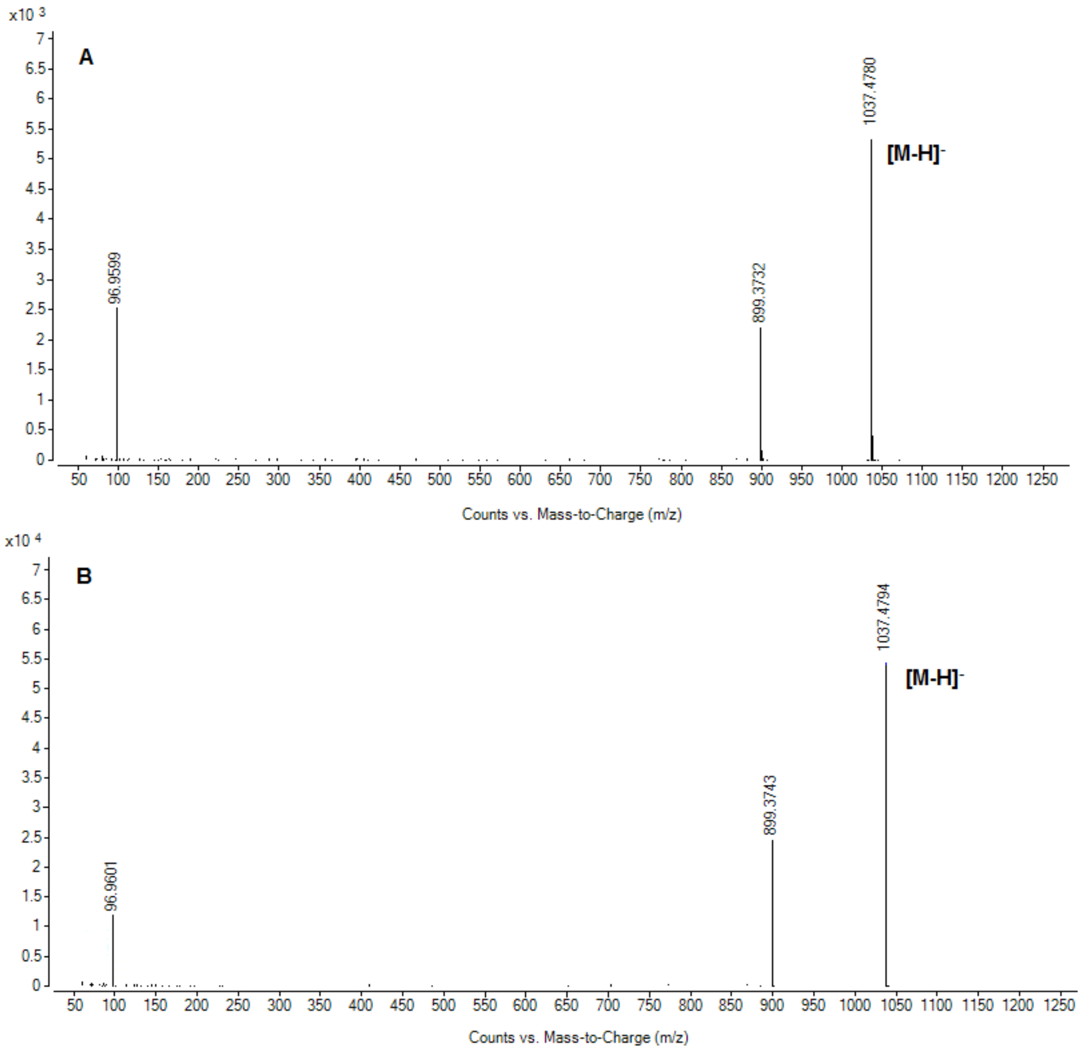

Figure S2. HRMS/MS spectra of [M-H]- (*m/z* 1037.4785) for (**A**) 44-methyl gambierone isomer at 6.0 min and for (**B**) 44-methyl gambierone at 6.6 min, resulting from an average of three collision energies (40, 65 and 90 eV).
